# Lack of Negatively Charged Residues at the External Mouth of Kir2.2 Channels Enable the Voltage-Dependent Block by External Mg^2+^

**DOI:** 10.1371/journal.pone.0111372

**Published:** 2014-10-28

**Authors:** Junwei Li, Xiaoxiao Xie, Jun Liu, Hui Yu, Suhua Zhang, Yong Zhan, Hailin Zhang, Diomedes E. Logothetis, Hailong An

**Affiliations:** 1 Institute of Biophysics, Hebei University of Technology, Tianjin, China; 2 Key Laboratory of Neural and Vascular Biology, Ministry of Education, Key Laboratory of Pharmacology and Toxicology for New Drug, Hebei Province, Department of Pharmacology, Hebei Medical University, Shijiazhuang, Hebei Province, China; 3 Department of Physiology and Biophysics, School of Medicine, Virginia Commonwealth University, Richmond, VA, United States of America; Xuzhou Medical college, China

## Abstract

Kir channels display voltage-dependent block by cytosolic cations such as Mg^2+^ and polyamines that causes inward rectification. In fact, cations can regulate K channel activity from both the extracellular and intracellular sides. Previous studies have provided insight into the up-regulation of Kir channel activity by extracellular K^+^ concentration. In contrast, extracellular Mg^2+^ has been found to reduce the amplitude of the single-channel current at milimolar concentrations. However, little is known about the molecular mechanism of Kir channel blockade by external Mg^2+^ and the relationship between the Mg^2+^ blockade and activity potentiation by permeant K^+^ ions. In this study, we applied an interactive approach between theory and experiment. Electrophysiological recordings on Kir2.2 and its mutants were performed by heterologous expression in *Xenopus laevis* oocytes. Our results confirmed that extracellular Mg^2+^ could reduce heterologously expressed WT Kir2.2 currents in a voltage dependent manner. The kinetics of inhibition and recovery of Mg^2+^ exhibit a 3∼4s time constant. Molecular dynamics simulation results revealed a Mg^2+^ binding site located at the extracellular mouth of Kir2.2 that showed voltage-dependent Mg^2+^ binding. The mutants, G119D, Q126E and H128D, increased the number of permeant K^+^ ions and reduced the voltage-dependent blockade of Kir2.2 by extracellular Mg^2+^.

## Introduction

Inwardly-rectifying potassium (Kir) channels play key physiological roles, such as in the control of heart rate, stabilization of the resting membrane potential and regulation of membrane excitability [1]–[5]. Kir channels are named for their ability to pass inward currents more easily than outward currents, a property known as inward rectification, which is the result of voltage-dependent block by cytosolic cations such as Mg^2+^ and polyamines [1], [4], [6], [7]. Kir channels are regulated by several factors, some of which are shared by family members (pH_i_, lipids), and some that are specific for subfamily members (nucleotides, G-protein, intracellular Na^+^ and extracellular K^+^) [8].

All Kir channels are tetrameric, with each subunit composed of cytoplasmic C- and N-terminal domains connected by two transmembrane helix domains (M1 and M2) that are linked by a P loop that forms the selectivity filter, a pore helix and a extracellular ‘turret’ loop (a re-entrant ‘turret’ loop). The selectivity filter at the extracellular mouth of the pore could also serve as a gating element [9]. Structural and computational evidence has shown that the K^+^ -channel selectivity filter consists of five binding sites (S0–S4) with 2–3 sites occupied at any given time, protecting against a collapse of the filter [10]–[14]. Specific residues in the outer mouth of the Kir channel might constitute a functional K^+^ sensor that could permit the channel to regulate its activity in response to changes in extracellular K^+^ [15]–[17]. Conduction through Kir2.1 is increased by negative surface charges at outer mouth of the pore originating from glutamate residues at position 153 [15]. Surface charges have also been shown to affect channel conductance in a variety of ion channels, such as neuronal Na^+^ channels [18], Ca^2+^ -activated K^+^ channel (BK) channels [19], and nicotinic acetylcholine receptors (NAChR) [20], presumably by influencing the concentration of permeant ions at the outer mouth [21].

It has been hypothesized that extracellular K^+^ interacts with Kir channels and subsequently increases channel open probability [22]–[24]. Direct activation of K channels by K^+^ has been proposed as an explanation for the increase in K^+^ channel activity (in various types of K^+^ channel) caused by increased [K^+^]_o_ [22]–[26]. Outward current of Kir2.1 is larger at higher [K^+^]_o_, because single-channel conductance is elevated at higher [K^+^]_o_ [27]. Kir1.1 channels are also activated by [K^+^]_o_ in the millimolar range [16], [28]. Mg^2+^ added to the extracellular solution reduced the amplitude of the single-channel currents of Kir1.1 channel [29], [30]. Biermans and colleagues (1987) showed that removing divalent cations from the external solution reduced the extent of inactivation of the inwardly rectifying K^+^ channels and silmilarly in heterologously expressed Kir1.1 [31], [32]. Blockage of Kir channels, such as Kir1.1, Kir2.1 or Kir3.1/3.4, by external Mg^2+^ was also reduced by increased extracellular K^+^ [29], [32], [33]. The effects of Mg^2+^ are antagonized by K^+^ in a manner which suggests that K^+^ competes with Mg^2+^ for an external inactivation site [30], [33]. However, the detailed mechanism by which permeant K^+^ ions elevate the function of K channels is not clear.

In this study, we identified that external Mg^2+^ can reduce the inward currents of Kir2.2 in a voltage-dependent way. Kir2.2 is one of two Kir mammalian channels (the other being Kir3.2) for which more complete crystal structures have been obtained for transmembrane and cytosolic domains [34]–[36]. MD (Molecular Dynamics) simulations show that one Mg^2+^ stays at the mouth of the selectivity filter, which causes a reduction of inward currents of Kir2.2. Through mutagenesis data and MD simulations we demonstrate that negative residues at the outer mouth of the pore collect permeant ions, i.e. K^+^, which reduce the voltage-dependent blockade of inward currents by extracellular Mg^2+^ by electrostatic repulsion.

## Materials and Methods

### Molecular Biology and preparation of *Xenopus oocytes*

Complementary DNA coding Kir2.2 was inserted into the pGEMHE plasmid vector. Site-specific mutants were produced by *Pfu* DNA polymerase with a QuickChange kit (Stratagene). Sequences were confirmed by DNA sequencing. cRNA was produced with T7 RNA polymerase using a kit (Ambion, Austin, TX). Recombinant Kir2.2 and its mutants were expressed in *Xenopus laevis* oocytes. cRNAs of the various Kir channels and their mutants were injected in the range of 0.5–1 ng/oocyte depending on the functional expression level of each channel.

The care of *Xenopus laevis* was in accordance with the guideline of the Institute of Experimental Animal Sciences, Hebei Medical University. The protocol was approved by the Animal Experiment Committee of Hebei Medical University. Ovarian lobes were dissected from adult female *Xenopus laevis* deeply anesthetized by ice for 30–40 min. A few lobes of ovaries were removed after a small abdominal incision (∼5 mm). The surgical incision was then sutured and the frogs were allowed to recover from anesthesia. The *Xenopus* oocytes were treated with collagenase (2 mg/ml, Sigma, Type II) in OR_2_ solution containing (in mM): 82.5 NaCl, 1 MgCl_2_, 2 KCl and 5 HEPES, pH 7.4 with NaOH. Oocytes were digested for 90–120 min at room temperature (∼24°C). Isolated oocytes were stored at 18°C in ND96 solution, which contains 96 mM KCl, 1 mM MgCl_2_, 1.8 mM CaCl_2_, HEPES 10 mM, sodium pyruvate 2.5 mM, and 50 mg/l Gentamicin. Experiments were performed with the wild-type and three mutant Kir2.2 channels. A Kir2.2 channel mutant, in which three outer vestibule amino acids (Gly119, Gln126 and His128) were changed to negatively charged residues, was studied in the *Xenopus laevis* oocyte system.

### Electrophysiology

Whole-cell current recordings in *Xenopus laevis* oocytes were performed 1–2 days after cDNA injection using a two-electrode voltage clamp. Current recordings were conducted at room temperature (22–24°C) using a GeneClamp 500B amplifier (Axon Instruments). Glass microelectrodes were filled with 3 M KCl dissolved in 1% agarose to prevent the leakage of KCl into the oocytes. The electrodes had a resistance less than 1 MΩ. Oocytes were bathed in a HK solution (in mM) (96 KCl, 1 MgCl_2_ and 10 HEPES, pH 7.4 with KOH) or HK-5EGTA (in mM) (96 KCl, 5 EGTA and 10 HEPES, pH 7.4 with KOH). Oocytes were clamped to a holding potential of −70 mV, and currents were recorded during 50-ms voltage steps ranging from −70 mV to +70 mV in 10-mV increments.

### Data analysis

All data acquisition and analysis were done with pClamp9.2, pClampfit9.2 (Axon Instruments) and Origi8.0 (OriginLab Corp) software. Each experiment shown or described was performed on 5–8 oocytes of the same batch. A minimum of 2–3 batches of oocytes was tested for each experiment shown. All data are shown as mean ± SEM. The degree of inhibition (γ) was calculated from the current according to the following equation γ = (I_0_–I_f_)/I_0_, where I_0_ is the initial current, I_f_ is final current.

### Homology modeling

The crystallographic structure of the chicken Kir2.2 (PDB code: 3JYC) [36] was used as a template for modeling human Kir2.2 (88% sequence homology to cKir2.2). The target sequence (hKir2.2) was taken from Genbank (http://www.ncbi.nlm.nih.gov/Genbank/). Modeling was done with SWISS-MODEL webserver [37]–[39]. The model was evaluated with QMEAN (The QMEAN4 score is a composite score consisting of a linear combination of 4 statistical potential terms (estimated model reliability between 0–1) [40]. The pseudo-energies of the contributing terms are given their Z-scores with respect to scores obtained for high-resolution experimental structures of similar size solved by X-ray crystallography) [40]. The QMEAN4 score was 0.557 and QMEAN Z-score was −3.507. The mutant channels were constructed by substituting the WT side chain with the specified side chains. Each model was compared to its template to verify that the modeling step had not significantly altered backbone and side chain conformations.

### Molecular Dynamics (MD) simulations

In preparation for the simulations, two K^+^ ions were placed at positions S1 and S3 and water molecules were placed at positions S0, S2, and S4 in the selectivity filter. The hKir2.2 channels [wild-type (WT) and four mutants] were embedded in a fully hydrated, 100 Å×100 Å palmitoyl-2-oleoyl-sn-glycerol-phosphatidylcholine (POPC) bilayer. The water molecules were described using the TIP3 model. We had built atomic models according to addition of different solvent. The systems were made electrically neutral by addition Mg^2+^ and Cl^−^ of 150 mM, K^+^ and Cl^−^ of 150 mM and Mg^2+^ of 1.5 mM, K^+^ and Cl^−^ of 150 mM, respectively (A total of ∼143,000 atoms). The solvated systems gradually relaxed using a standard procedure: the entire protein and solution were fixed for 5 ns, enabling reorganization of the lipid; the solvent and the lipids equilibrated for 5 ns with only the protein restrained; finally the backbone atoms of cytoplasmic domain and two transmembrane helices (residues N44-A106, F134-L148, V159-A372) were only restrained with a harmonic constant k = 2.0 kcal/(mol Å), which was maintained to limit structural changes within this part of protein, though side chains, extracellular domain (residues V107-A133, R149-A158) and solution were left completely free for 15 ns.

MD simulations with explicit solvent and different ions were carried out on a total of 7 separate systems of hkir2.2 and mutants using NAMD [41] (http://www.ks.uiuc.edu/) with the CHARMM27 force field [42]. All simulations had the following characteristic: (a) Three-dimensional periodic boundary conditions were applied and long-range electrostatic forces were calculated using the particle mesh Ewald (PME) [43], [44] approach (108×108×200 grid points) with a grid spacing of ∼1 Å in each dimension; (b) The Langevin dynamics scheme was used to keep the temperature constant at 310 K. The Nosé-Hoover-Langevin piston was used to maintain the constant pressure at 1 atm; (c) The cutoff radius for Lennard Jones interactions was 12 Å, with a smooth switching function starting at 10 Å and a nonbonded ‘‘pairlist’’ distance of 13.5 Å; (d) A time-step of 2 fs was used to integrate the equations of motion and a reversible multiple time-step algorithm of 4 fs was used for the electrostatic forces and 2 fs for short-range, nonbonded forces. Overall, ∼175 ns of simulation time was generated and ∼155,000 CPU hours were used in calculating the results in this study.

The time series of the (*x,y,z*) position of all ions were generated using Visual Molecular Dynamics (VMD) [45]. To calculate the densities, the radial position was calculated as *R  = * (*x^2^+ y^2^*)^1/2^ at every frame. The time series was then binned into 1 Å increments of Δ*R* and ΔZ. The number of counts in each bin was divided by the total number of frames in the time series and by the volume element because the radial volume increases as *R* increases. The volume element is the cylindrical annulus calculated at the center of the *i* ^th^ bin as *ΔV_i_  =  π* (*R_i+1_^2^+ R_i_^2^*) *Δ*Z. The resultant densities are in units of ions/Å^3^.

Molecular visualization, system setup and analysis were done with the VMD software package.

## Results

### Extracellular Mg2+ reduces the inward currents of Kir2.2, but not Kir2.1, in a voltage dependent way

**Figure 1:**
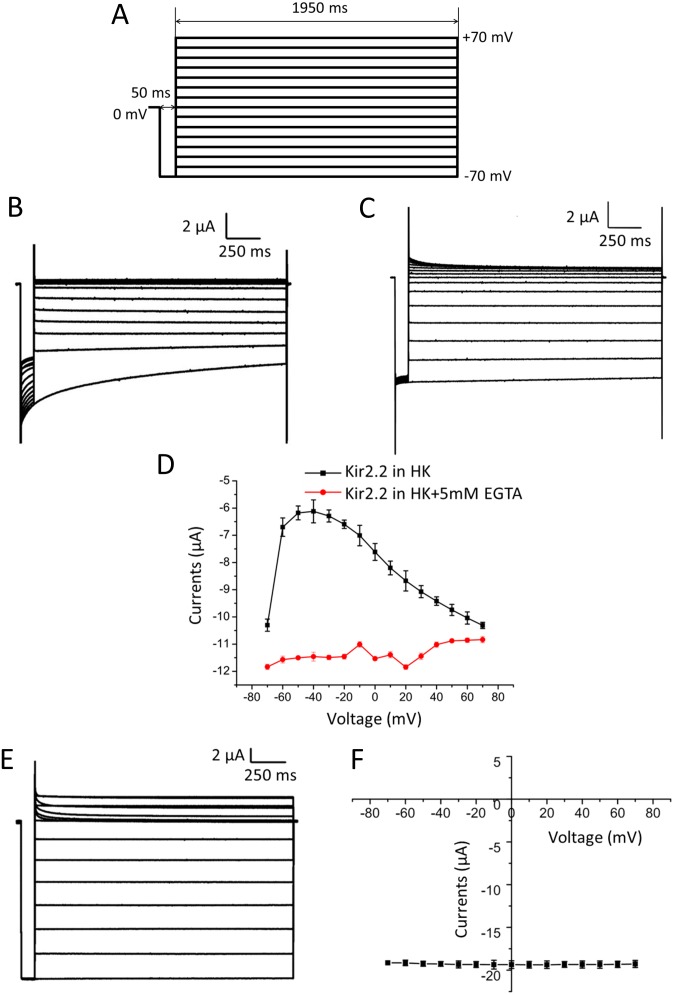
External Mg^2+^ voltage-dependently blocks the currents through Kir2.2 but not Kir2.1. Whole-cell currents were recorded by two-electrode voltage clamp. (A) Protocol used for activating the currents. The protocol contains a series of 2s-long sweeps. Each sweep consisted of a 50 ms test pulse to −70 mV and 1950 ms conditional pulse from −70 mV to +70 mV in 10 mV steps. There are 15 sweeps for each experiment. The first 50-ms test pulse (−70 mV) is going before the conditional pulses. As a control, the test pulses follow the conditional pulses. (B) and (C) are the representative currents Kir2.2 recorded with and without Mg^2+^. (D) is the relationship between the currents of Kir2.2 recorded at −70 mV and the corresponding conditional voltage. The data recorded with 1 mM Mg^2+^ and 0 Mg^2+^ are shown as black and red lines, respectively. (E) is the representative currents Kir2.1 recorded with Mg^2+^. (F) is the relationship between the currents of Kir2.1 recorded at −70 mV and the corresponding conditional voltage. Data represent means ± SEM for 7 experiments.

In these experiments, whole-cell currents were recorded from oocytes expressing either WT or mutants of Kir2.2 channels using two-electrode voltage clamp. Starting with a 50-ms test pulse to −70 mV, while conditioning pulses from −70 mV to 70 mV were applied to the oocytes expressing Kir2.2 channel in steps of 10 mV (2s duration). There are 15 sweeps for each experiment. The first 50-ms test pulse (−70 mV) is going before the conditioning pulses. As a control, the test pulses follow the conditioning pulses (Fig. 1A). Evoked by this protocol, the currents of Kir2.2 at various test potentials showed a voltage dependent inhibition with 1 mM extracellular Mg^2+^ (Fig. 1B and D). The inhibited currents reached peak values after the oocytes were held for 2 s at −70 mV. Removing the Mg^2+^ in bath solution by 5 mM EGTA also eliminated voltage-dependent inhibition of Kir2.2 channels, indicating that it was the Mg^2+^ that was responsible for the voltage-dependent reduction of the Kir2.2 current (Fig. 1C and D). Under the same experimental conditions, whole-cell currents of Kir2.1 were also measured. There were no voltage dependent changes observed for Kir2.1 currents (Fig. 1E and F). From these results we concluded that external Mg^2+^ could inhibit inwardly rectifying currents of Kir2.2 channels in a voltage-dependent way.

### External Mg^2+^ ions bind to the Kir2.2 channel to block inward currents

**Figure 2:**
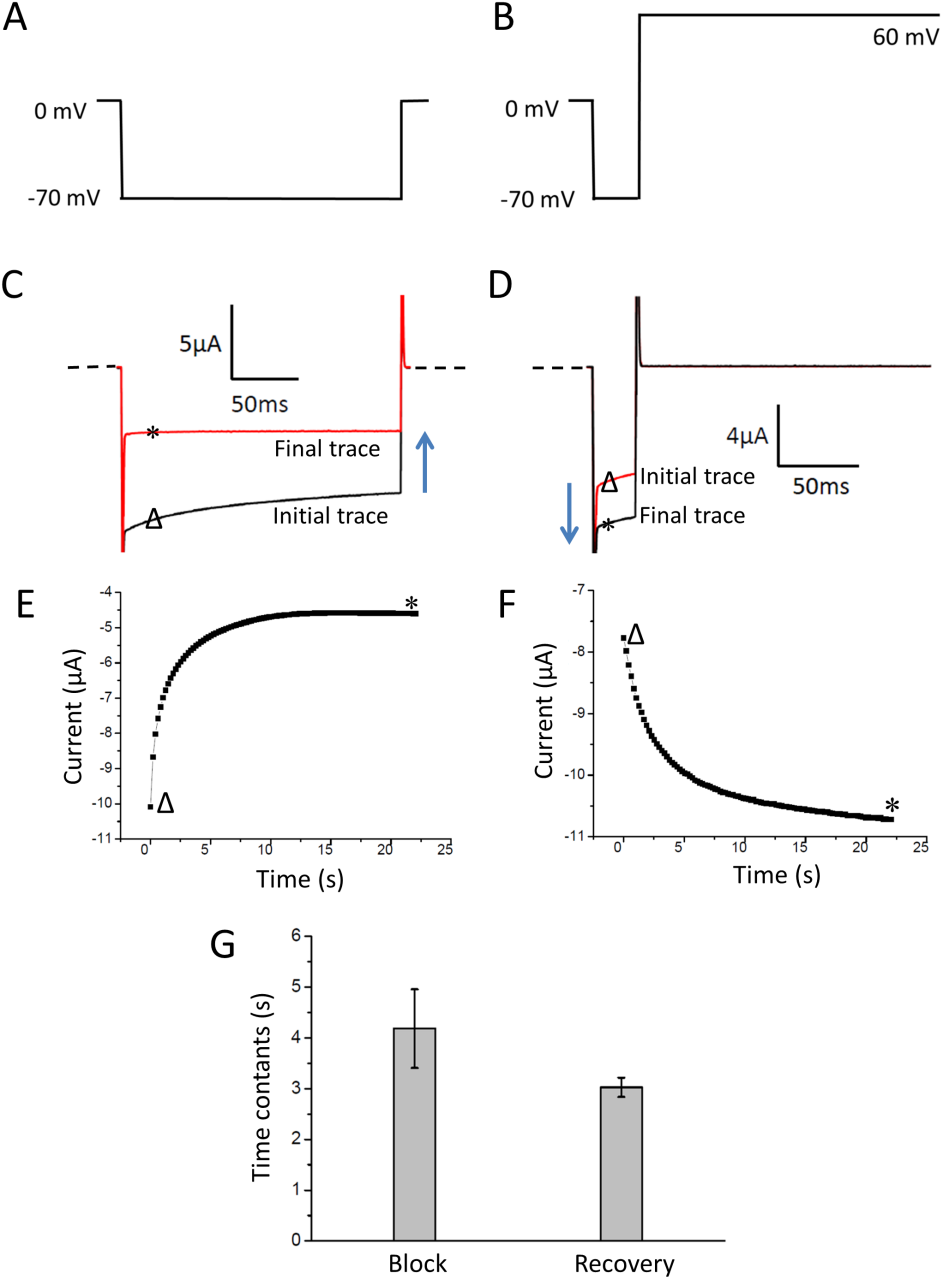
The kinetics of inhibition and recovery induced by Mg^2+^ wash-in or wash-out. (A) and (B) are the protocols used to inhibit, activate the currents, respectively. The protocols will repeat until the currents reach the plateau. (C) and (D) are the representative current traces of inhibition and recovery processes. Δ and * show the currents of initial and final traces. (E) and (F) are the inhibition and recovery kinetics. Δ and * show current corresponding to C and D. (G) shows the summary data of the time constants corresponding to E and F. Data represent means ± SEM for 7 experiments.

To further investigate the mechanism of inhibition of Kir2.2 currents by extracellular Mg^2+^, we analyzed the kinetics of the current inhibition and recovery processes. Figure 2A, C and E show the protocol, current inhibition and inhibition kinetics induced by wash-in of external Mg^2+^ ions at −70 mV. Figure 2B, D and F show the protocol, current recovery level and kinetics when the membrane was held at positive 60 mV. Both the inhibition and recovery show comparable time constants 4 s and 3 s, respectively (Fig. 2G). We speculated that the external Mg^2+^ could bind directly to the outer mouth of the Kir2.2 channel to cause the rapid inhibition of the inward K^+^ current.

### Binding site of extracellular Mg^2+^

**Figure 3:**
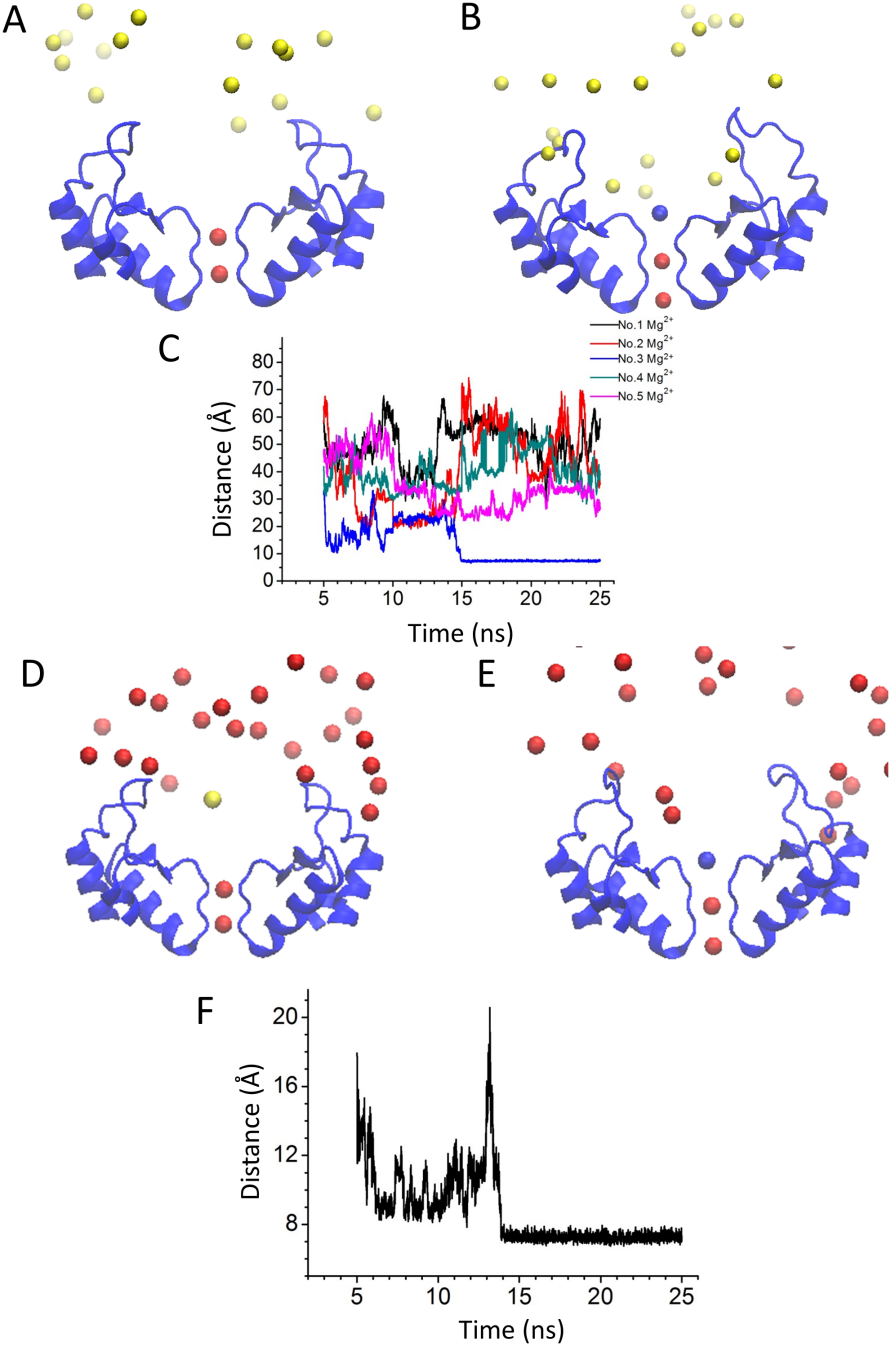
Mg^2+^ ion binds at the S0 site of Kir2.2 both in high and physiological conditions. Distributions of extracellular K^+^ and Mg^2+^ ions of the initial (A and D) and final (B and E) snapshots. Mg^2+^ and K^+^ ions are represented by yellow and red spheres, respectively. In panel B and E, the blue spheres represent the binding Mg^2+^ ions. (C and F) Evolution of the distance (between the center of the selective filter and each Mg^2+^) against time (5–25 ns). The total simulation time was 25 ns. In the first 5 ns, the ions are fixed for equilibration of the lipids. The No. 3 Mg ^2+^ is corresponding to the bound Mg^2+^.

**Figure 4:**
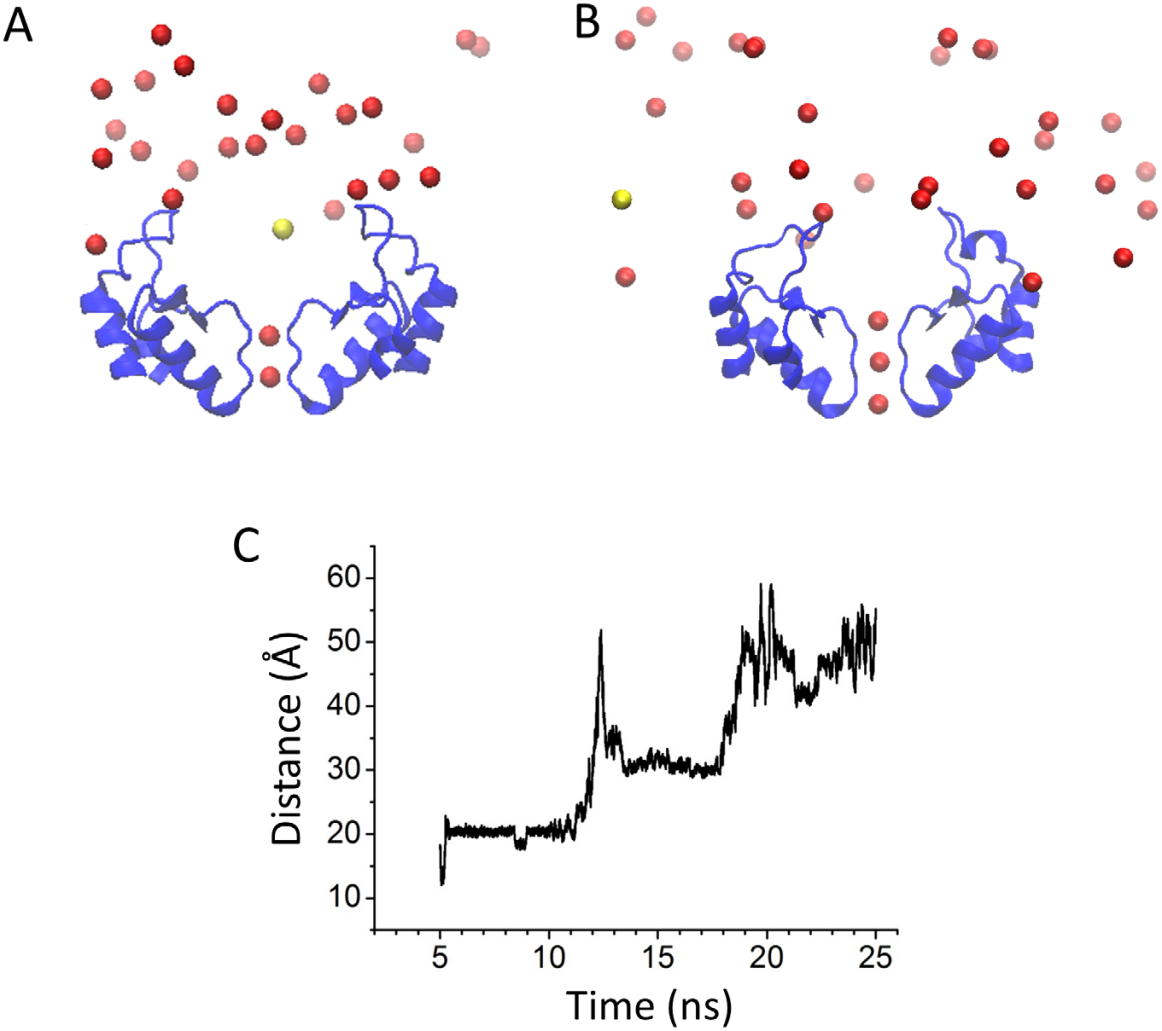
K^+^ ions compete with Mg^2+^ to bind at the S0 site. Distributions of extracellular K^+^ and Mg^2+^ ions of the initial (A) and final (B) snapshots. Mg^2+^ and K^+^ ions are represented by yellow and red spheres, respectively. (C) Evolution of the distance (between the center of the selective filter and Mg^2+^) against time (5–25 ns). The total simulation time was 25 ns. In the first 5 ns, the ions are fixed for equilibration of the lipids.

**Figure 5:**
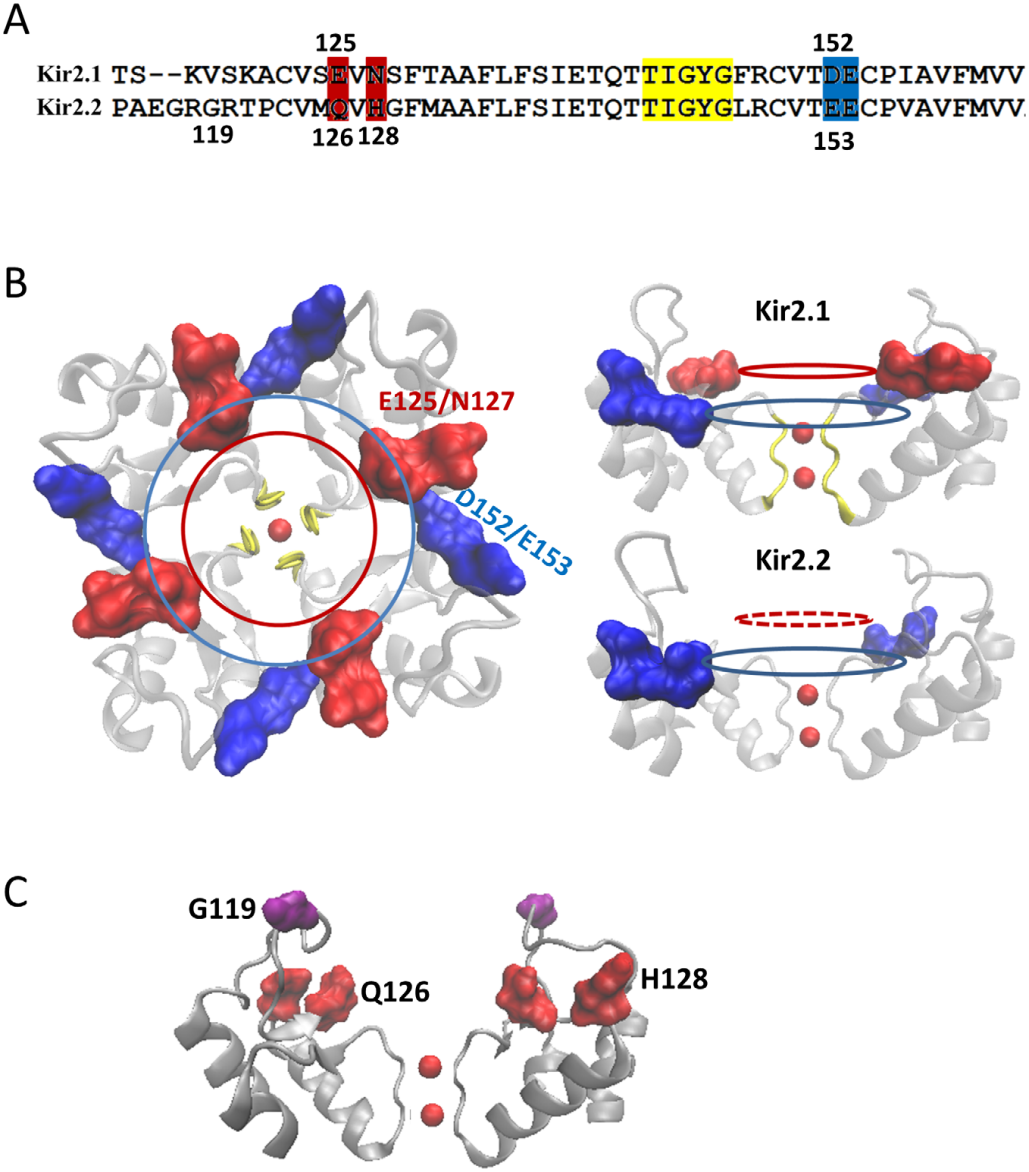
There is only one negative ring in Kir2.2 but two in Kir2.1. Sequence alignments for Kir2.1 and Kir2.2 (A) and the schematic positions of the negative rings (B). There are two negative rings in Kir2.1 formed by three negatively charged and one polar amino acid side chains which are E125 & N127 (highlighted in red circle), D152 & E153 (highlighted in blue circle). Whereas, Kir2.2 channel lacks one of the two negatively charged rings (red dotted line circle, bottom right). For clarity, only two of four subunits are shown. (C) Ribbon model of the extracellular side of two subunits of Kir2.2 showing the positions of G119 (purple), Q126 (red) and H128 (red).

To determine the putative Mg^2+^ binding sites, we performed five Molecular Dynamics (MD) simulations with the Kir2.2 channel. One of Simulations were carried out in 150 mM MgCl_2_. Figure 3A shows the initial structure in which the Mg^2+^ ions randomly scatter at the outside of the Kir2.2 channel. During this MD simulation, the external Mg^2+^ ions were rearranged. One of the Mg^2+^ ions moved to and was stabilized at the mouth of the selectivity filter (SF) (Fig. 3B, and C), which could block the channel and reduce K^+^ inward current. We carried out another four simulations of the Kir2.2 channel in ∼150 mM K^+^, with ∼1.5 mM Mg^2+^ at the outside membrane (Fig. 3D and 4A). After the equilibrated bulk solution, the external ions were rearranged. There are two of four simulations in which the Mg^2+^ can bind at S0 binding site of the outer mouth of the pore (Fig. 3E, and F). The other two simulations show that one of the K^+^ occupied the Mg^2+^ binding site at S0 site (Fig. 4). Our data show that in physiological concentration, Mg^2+^ and K^+^ compete to bind at S0 site. These results suggested that entry to the external mouth of the channel pore could underlie the extracellular Mg^2+^ blockade. To determine potential key residues which could mediate the voltage-dependent blockade of Kir2.2 by Mg^2+^ ions, we aligned the amino acid sequences (Fig. 5A) and compare the 3D structures (Fig. 5B) of Kir2.1 and Kir2.2. As shown in Figures 5A and B, there are two sets of negatively charged residues in Kir2.1 forming two concentric rings (seen from the outside to the inside as E125-N127 and D152–E153). However, in Kir2.2 there is only one negatively charged ring in the outer mouth of SF in Kir2.2 (Fig. 5B), with residues Q126 and H128 in place of the Kir2.1 E125 and N127 (Fig. 5A and C). The structure suggested that the Kir2.2 (G119) residue is positioned on top of the Q126 and H128 residues. To examine whether the negative charge of the Kir2.1 (E125) caused the difference between the Kir2.1 and 2.2 in the Mg^2+^ -induced voltage-dependent block, we designed three single mutants of Kir2.2, G119D, Q126E, H128D and the triplet mutant (G119D, Q126E and H128D).

### Mutants weaken the voltage-dependent blockage of Kir2.2 by external Mg^2+^

**Figure 6:**
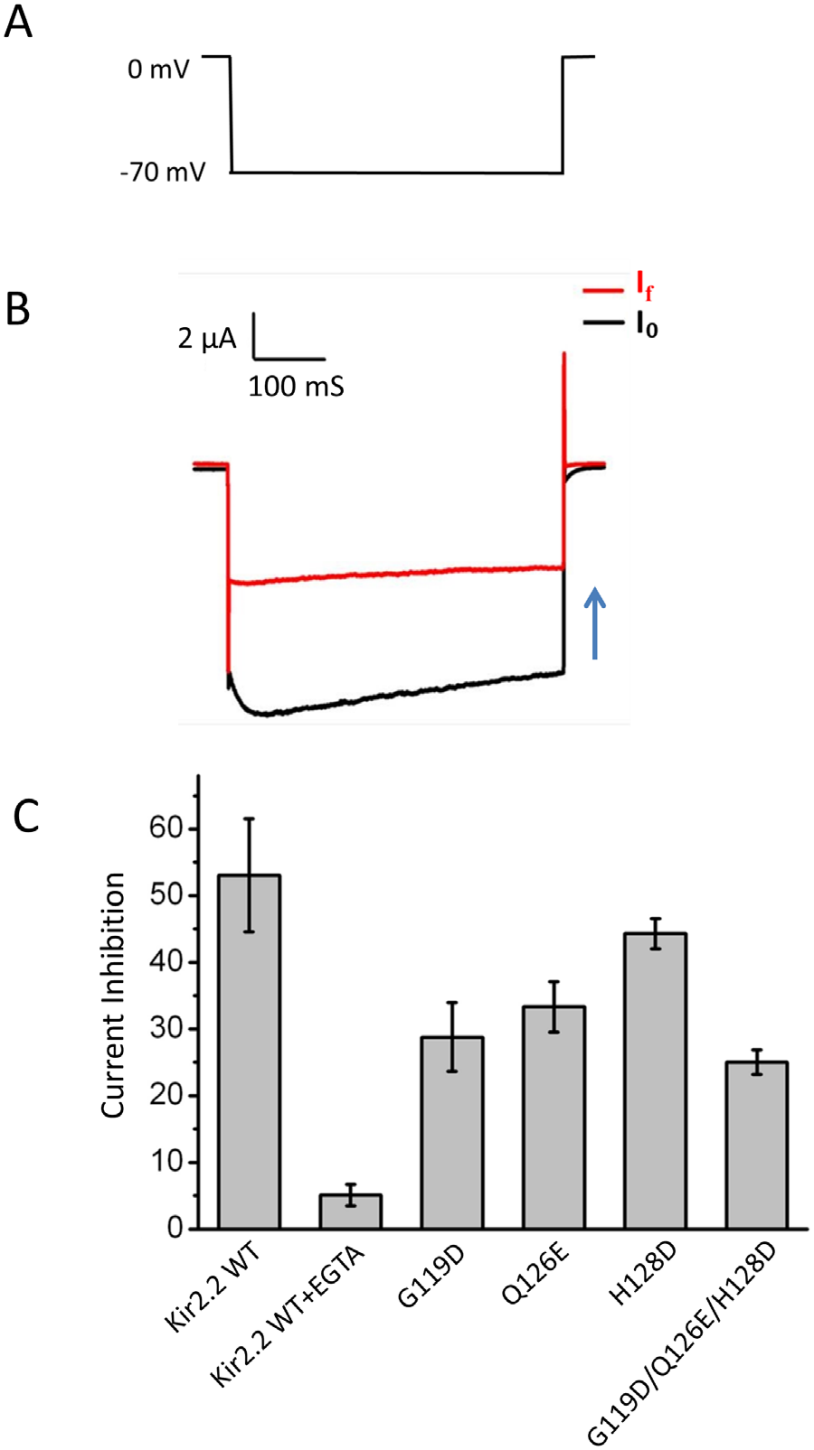
The mutants of Kir2.2 weaken the external Mg^2+^ blockage. (A) The protocol used for recording the degree of inhibition which was hyperpolarization from 0 mV holding potential to −70 mV for 500 ms, and then stepped to 0 mV. (B) The representative currents trace in Kir2.2 channel. (C) degree of Inhibition (γ = (I_0_–I_f_)/I_0_) for wild type (WT), WT+EGTA, G119D, Q126E, H128D and Triplet mutant (G119D, Q126E and H128D) channels.

Next, we tested whether mutations of the residues suggested by the MD simulations affected the voltage-dependent block of Kir2.2 currents by external Mg^2+^. We defined the degree of inhibition (γ) as γ = (I_0_–I_f_)/I_0_, where I_0_ and I_f_ correspond to the initial and final currents of Kir2.2 or its mutants. The Mg^2+^-induced degree of inhibition (γ) of inward K^+^ current at −70 mV (Fig. 6A and B) was assessed. Figure 6C shows that the degree of inhibition of WT, G119D, Q126E, H128D and the triple mutant (G119D, Q126E and H128D) induced by extracellular Mg^2+^ were (54±8)%, (29±5)%, (33±3)%, (44±2)% and (25±2)%, respectively. These data showed that the mutants significantly diminished the voltage-dependent block of Kir2.2 by external Mg^2+^.

### Mutations increase the collection of K^+^ ions at the outer mouth of the SF

**Figure 7:**
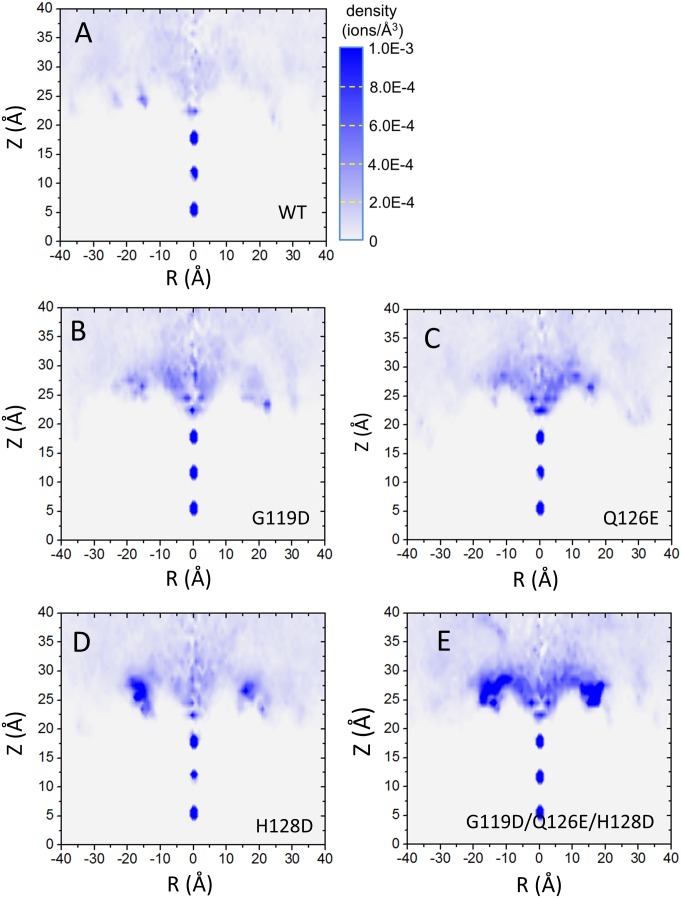
Extracellular K^+^ density distribution in Kir2.2 and its mutants. The simulations of the Kir2.2 and its mutants were carried out in 0.15 M K^+^. The extracellular K^+^ density plots are shown as functions of radial distance from the pore axis (*R = *(*x^2^+y^2^*)^1/2^) and height in the simulation box (Z). K^+^ density distribution in Kir2.2 WT (A), G119D (B), Q126E (C), H128D (D) and Triplet mutant (G119D, Q126E and H128D) channels (E).

To better understand how the outer ring mutants decreased the Mg^2+^-induced block of inward K^+^ current in Kir2.2, we performed MD simulations on the WT and the mutants of Kir2.2 channel with randomized initial configurations of 0.15 M KCl. Figure 7 shows density plots of the WT (Fig. 7A) single mutants, G119D, Q126E, H128D (Fig. 6B–D, respectively) and the triple mutant (G119D, Q126E and H128D) (Fig. 7E), revealing that the negatively charged mutants (especially the triple mutant) increased the number of permeant K^+^ ions at the outer mouth of the channel. The density analysis is consistent with the electrophysiological data (see Fig. 6). These results lead us to conclude that the negatively charged residues at the outer mouth of Kir2.2 channel increase the density of permeant K^+^ ions at the outer mouth of the selectivity filter which repel Mg^2+^ from binding the channel reducing its block of inward K^+^ current.

## Discussion

### Inward currents of Kir2.2 but not Kir2.1 show Mg^2+^-induced voltage-dependent inhibition

In this study, we identified that external Mg^2+^ ions can inhibit inward currents through Kir2.2 but not Kir2.1 in a voltage-dependent manner. However, our data is different from a previous study Murata and colleagues [46], which showed that external Mg^2+^ Kir2.1 currents but had weak effects on Kir2.2 and Kir2.3 currents. Murata et al. tested the effect for external Mg^2+^ on Kir2.1, Kir2.2 and Kir2.3 and showed that the extracellular Mg^2+^ could reduce the inward currents of Kir2.1 in a dose-dependent manner. They recorded the currents with a step-pulse protocol, from −150 to +20 mV in 10 mV increments for 0, 1, 3, 10 mM [Mg^2+^]_o_. There was significant inhibition at −150 mV but a very weak effect at −70 mV of Kir2.1. We focused on the inhibition of the inward currents of Kir2.2 held at −70 mV with 1 mM [Mg^2+^]_o_.

### Extracellular Mg^2+^ decreases affinity for K^+^ by binding with the channel

Intracellular cations, such as Mg^2+^ and polyamines, can block the outward currents of Kir channels under depolarized membrane potentials, a phenomenon referred to as inward rectification [4]. In this study, we report that under polarized membrane potentials, physiological concentrations of external Mg^2+^ (1 mM) reduced the inward currents of Kir2.2, but not Kir2.1. Depolarized potentials facilitated channel recovery from the Mg^2+^-induced block. Our findings suggest that the block of extracellular Mg^2+^ on inward currents through Kir2.2 channels displays voltage dependence. As reported by a previous study, the extracellular divalent cations (Ca^2+^ and Mg^2+^) can increase inactivation of Kir1.1 [32] and reduce the single-channel currents through Kir1.2 channels [29]. However, the detailed molecular mechanism of the regulation of Kir channels by external cations is not known. Extracellular divalent cation (Ca^2+^, Ba^2+^ and Mg^2+^) interaction with the outer mouth of the pore, just external to the selectivity filter, have been reported to decrease Kir currents [47], [48]. Following the idea that the Mg^2+^ ions may bind at the extracellular mouth of the channel, reducing the affinity of the channel for K^+^ and thus block K^+^ currents through Kir2.2, we performed MD simulations and found that Mg^2+^ ions can be stabilized at the external mouth of the Kir2.2 channel.

We found that differences in the voltage-dependent Mg^2+^ block between different Kir channels may be due to extracellular negatively charged amino acid residues present at the external mouth of the pore (Fig. 5A). The negatively charged residue (E153) contributes to the conductance properties of Kir2.1 channels by acting as a surface charge [15]. In the presence of divalents, the E125Q and E153C mutants of Kir2.1 channels showed decreased single-channel conductance compared to the WT channels [15], [46]. The negative charges of Kir1.1 channel stabilize external K^+^ in the selectivity filter or at the S0-K binding site just outside the filter [32]. The negatively charged residue (E125) in an extracellular loop of Kir2.1 facilitates K^+^ permeation as described previously, the peak currents reduced from 25 to 5 µA by introducing the E125Q mutation [46]. Our MD simulation results demonstrate that the extracellular negatively charged residues can enhance the external K^+^ accumulation at the outer mouth of the Kir2.2 pore (Fig. 7).

It has been reported that there is interactions between the extracellular blocker Mg^2+^ and the permeant ions K^+^. Our results agree with previous reports by showing that extracellular surface negatively charged residues relief the blockage of Mg^2+^_o_ by increasing the [K^+^]_o_. Extracellular Mg^2+^ can reduce single-channel currents with an affinity that increased as [K^+^]_o_ decreased [29]. The sensitivity to the block by Mg^2+^_o_ was increased by lowering extracellular K^+^ (K^+^_o_), suggesting a competitive interaction of Mg^2+^_o_ and K^+^_o_ in the Kir2.1 channel [46]. Increased extracellular K^+^ concentration can inhibit the block of Mg^2+^_o_. With Kir2.1 and Kir3.1/Kir3.4, decreasing the extracellular K^+^ concentration augmented the block which caused by external divalent cations (Ba^2+^, Mg^2+^ and Ca^2+^) [33]. The magnitude of current reduction of Kir1.1 by external Mg^2+^ increases as the extracellular K^+^ concentrations decreases [47]. The elimination of a negative charge in the outer mouth of Kir1.1 might decrease Mg^2+^ and/or K^+^ affinities for the channel through favorable electrostatic interactions [47].

We conclude proposing a possible mechanism for the block of Kir2 channels by extracellular Mg^2+^. In this mechanism Mg^2+^ is stabilized at the external mouth of the Kir2 channel to inhibit K^+^ entry. This effect is likely to be by direct block of the current rather than an allosteric conformational change. The proposed model is consistent with the relationships between the external Mg^2+^, K^+^ and the negatively charged outer mouth of pore. Augmentation of extracellular K^+^ concentration can decrease the block of Kir channel by extracellular Mg^2+^ by electrostatic repulsion.
